# Early exposure to UV radiation causes telomere shortening and poorer condition later in life

**DOI:** 10.1242/jeb.243924

**Published:** 2022-09-02

**Authors:** Niclas U. Lundsgaard, Rebecca L. Cramp, Craig E. Franklin

**Affiliations:** School of Biological Sciences, The University of Queensland, Brisbane, QLD 4072, Australia

**Keywords:** Amphibian, Carryover effect, Irradiance, Life-history trade-off, Telomere length, Ultraviolet radiation

## Abstract

Determining the contribution of elevated ultraviolet-B radiation (UVBR; 280–315 nm) to amphibian population declines is being hindered by a lack of knowledge about how different acute UVBR exposure regimes during early life-history stages might affect post-metamorphic stages via long-term carryover effects. We acutely exposed tadpoles of the Australian green tree frog (*Litoria caerulea*) to a combination of different UVBR irradiances and doses in a multi-factorial laboratory experiment, and then reared them to metamorphosis in the absence of UVBR to assess carryover effects in subsequent juvenile frogs. Dose and irradiance of acute UVBR exposure influenced carryover effects into metamorphosis in somewhat opposing manners. Higher doses of UVBR exposure in larvae yielded improved rates of metamorphosis. However, exposure at a high irradiance resulted in frogs metamorphosing smaller in size and in poorer condition than frogs exposed to low and medium irradiance UVBR as larvae. We also demonstrate some of the first empirical evidence of UVBR-induced telomere shortening *in vivo*, which is one possible mechanism for life-history trade-offs impacting condition post-metamorphosis. These findings contribute to our understanding of how acute UVBR exposure regimes in early life affect later life-history stages, which has implications for how this stressor may shape population dynamics.

## Abstract (precis)

**Summary:** Ultraviolet radiation exposure in amphibian larvae generates detrimental carryover effects on body condition and relative telomere length post-metamorphosis, a mechanism that may influence amphibian population dynamics.

## INTRODUCTION

Solar ultraviolet-B radiation (UVBR; 280–315 nm) is a high-energy electromagnetic radiation and a pervasive stressor for many organisms (Williamson et al., 2019). In addition to facilitating endogenous vitamin D_3_ synthesis (Antwis and Browne, 2009), this genotoxic stressor can also form pyrimidine dimer lesions in DNA that disrupt transcription and replication, which can in turn lead to cancer, cell apoptosis and tissue damage (Batista et al., 2009; Londero et al., 2019). UVBR also causes oxidative stress through the production of reactive oxygen species (ROS; Heck et al., 2003; Kazerouni et al., 2016), although ultraviolet-A radiation (UVAR; 315–400 nm) is more potent in this regard (Schuch et al., 2017). Despite organisms having DNA repair mechanisms to remove UVBR-induced DNA damage (Sancar and Sancar, 1988; Sancar and Tang, 1993), even minor increases in UVBR irradiance may be sufficient to tip DNA damage rates beyond the capacity for repair, which can have detrimental downstream effects on organismal condition and survival (Pandelova et al., 2006; Schuch et al., 2015b).

Stratospheric ozone depletion and changes in climate have caused widespread increases in the irradiance and fluctuation of UVBR in regions of amphibian decline (Farman et al., 1985; Kerr and McElroy, 1993; Herman et al., 1996; Middleton et al., 2001; Schuch et al., 2015a). Ambient UVBR can have a range of lethal and sublethal effects across many amphibian species (Blaustein et al., 1995, 2005; Tietge et al., 2001; Bancroft et al., 2008) and these effects may be exacerbated by interactions with other factors including disease, pollutants and temperature (Blaustein et al., 2003; Bancroft et al., 2008; Alton and Franklin, 2017; Cramp and Franklin, 2018; Lundsgaard et al., 2020). However, two significant gaps in this body of literature hinder the effective extrapolation of these individual-level effects of UVBR to population-scale effects and declines. Firstly, it is not known which parameters of UVBR exposure determine amphibian health risk, be it irradiance, dose or exposure duration (Lundsgaard et al., 2021), which leads to discrepancies in the estimated risks posed to amphibians by field measurements of UVBR (Palen et al., 2002; Blaustein et al., 2004; Olker et al., 2013). Secondly, most research has focused on early life-history stages which are most susceptible to UVBR exposure, but that do not strongly drive population dynamics (Vonesh and De la Cruz, 2002; Vonesh, 2005). Post-metamorphic life-history stages have the strongest influence on amphibian population dynamics (Biek et al., 2002) but are typically overlooked in UVBR research because of their mainly nocturnal habits. What is rarely considered are potential physiological carryover effects that link embryonic and larval UVBR exposure to impacts on post-metamorphic life stages.

Carryover effects are consequences of an early development experience that persist for a time after the experience that causes them ceases, such that cause and effect are separated by a measurable transitional period (O'Connor et al., 2014). For example, environmental factors encountered by embryonic and larval amphibians, including contaminants, predation, aquatic pH, food availability and conspecific density, have been shown to affect traits including locomotion, morphology, growth and survival in later life-history stages (Merila et al., 2000; Relyea, 2001; Rasanen et al., 2002; Vonesh, 2005; Chelgren et al., 2006; Tejedo et al., 2010; Van Allen et al., 2010; Boes and Benard, 2013; Touchon et al., 2013; Rumrill et al., 2018). Only a few studies have explicitly tested carryover effects of UVBR exposure in amphibians, with detrimental effects of exposure during embryonic and larval stages oftentimes only manifesting in later life-history stages (Smith et al., 2000; Pahkala et al., 2001; 2003; Belden and Blaustein, 2002; Ceccato et al., 2016; Lundsgaard et al., 2021). Such ‘latent effects’ are becoming increasingly apparent across taxa (Pechenik, 2006), yet little is known about the mechanistic basis for them, with changes in energy balance, oxidative stress, epigenetic modifications and telomere lengths all implicated (O'Connor et al., 2014; Young, 2018).

Telomeres are non-coding DNA sequence repeats (TTAGGG in vertebrates) on the ends of chromosomes (in eukaryotes) and serve a protective role in maintaining genome stability (O'Sullivan and Karlseder, 2010). In metazoans, this highly conserved nucleoprotein structure is shortened during each cellular division cycle, and can also be damaged by oxidative stress, potentially accelerating the shortening process (Young, 2018). Upon reaching a critical minimum length, replicative senescence is triggered to prevent mutation and cancer (Campisi and d'Adda di Fagagna, 2007). For these reasons, telomere length is a good proxy for long-term organismal health and longevity (Shalev et al., 2013). Given that telomere length is influenced by environmental stress and predicts long-term condition, it is likely that telomere shortening may be involved in the generation of life-history trade-offs and carryover effects (Young, 2018). To our knowledge, no study has investigated how UVBR exposure in early life might influence telomere length later in life. Improving understanding of how the dose, irradiance and duration of UVBR exposure interact on such carryover effects is crucial for elucidating the impacts of complex and increasing natural UVBR exposures on long-term amphibian health (Hegglin and Shepherd, 2009; Mckenzie et al., 2011).

Our aim was to investigate how acute exposure of larvae to different doses and irradiances of UVBR affects size, condition, performance and relative telomere lengths post-metamorphosis. We acutely exposed tadpoles of the Australian green tree frog, *Litoria caerulea*, to a combination of different UVBR irradiances and doses in a fully factorial laboratory experiment, and then reared these larvae to metamorphosis in the absence of UVBR so that there was a defined temporal gap between UVBR exposure and the physiological carryover effects being measured in the juvenile frogs upon development. UVBR irradiance determines the rate of DNA lesion formation (Londero et al., 2019), so we hypothesised that high irradiance UVBR would hinder successful metamorphosis, and would be more detrimental to growth, jumping performance and foraging efficiency in juvenile frogs than an equivalent dose of low irradiance UVBR. Given that animals exposed to high irradiance UVBR are expected to experience increased oxidative stress which can shorten telomeres, we also hypothesised that this treatment would cause shorter relative telomere lengths in metamorphs.

## MATERIALS AND METHODS

### Ethics statement

This research was approved by The University of Queensland Animal Ethics Committee (approval no. SBS/089/19 and 2021/AE000365) and animal collection permission was granted by the Queensland Department of Environment and Science (permit no. WISP17421516).

### Study species

*Litoria caerulea* (White 1790) is a common species distributed throughout northern and eastern Australia. The IUCN lists this species as ‘Least Concern’; however, population declines have been documented in some regions (Berger et al., 1998; http://www.iucnredlist.org). *Litoria caerulea* is relatively resilient to UVBR (Lundsgaard et al., 2021), which is consistent with its ecology, laying transparent gel egg masses in unsheltered, ephemeral water bodies that are exposed to UVBR levels of up to 500 µW cm^−2^ (at water surface: van Uitregt et al., 2007).

### Animal collection and husbandry

Seven freshly laid *L. caerulea* egg masses were collected from flooded roadsides in southeast Queensland, Australia, and transported to The University of Queensland in plastic bags containing water from the collection site. Each clutch was maintained in a separate 5 l plastic tank of carbon-filtered Brisbane city tap water and housed at 22.5±1°C on a 12 h light:12 h dark photoperiod regime using non-UVBR fluorescent lights. At the commencement of UVBR exposure treatments 4 weeks post-laying, a random selection of larvae of known clutch identity (Gosner stage 25; Gosner, 1960) were individually housed in clear plastic containers (1 l; 15×10×7 cm) filled to a depth of 5 cm with carbon-filtered Brisbane city water (see ‘Experimental Design’, below). Larvae remained in these containers during and following UVBR treatment exposure and were fed to satiation with thawed spinach throughout larval development, with half water changes (of carbon-filtered Brisbane city water) made twice per week to maintain water quality. When larvae developed front legs (Gosner stage 42), containers were partially emptied of water and angled such that metamorphosing animals could climb out of the water. Newly metamorphosed juvenile frogs were held individually in their original housing containers with perforated lids and soaked paper towel added as substrate. Frogs were fed to satiation with crickets and cockroaches (reared on a diet of carrot and dry cat food rich in essential vitamins and minerals) every fourth day until completion of tests 1 month post-metamorphosis, after which they were euthanised in a buffered Tricaine-S bath (MS-222; Aqua-Life, Nanaimo, BC, Canada; 0.5 g l^−1^). Prey items were not dusted in a dietary supplement powder because newly metamorphosed frogs showed little interest in consuming prey that was dusted.

### Experimental design

The experimental design employed in this study is also described in Lundsgaard et al. (2021), which reported on an earlier phase of this larger experiment. Four-week-old individually housed larvae (*n*=160) were randomly allocated to one of 10 UVBR treatments in a 3×3 factorial design (plus a no-UVBR treatment; *n*=16 per treatment), whilst ensuring that the distribution of clutch identity was consistent across treatments. Three levels of UVBR irradiance (low: 8.7 µW cm^−2^, medium: 35.5 µW cm^−2^, and high: 70 µW cm^−2^; Table S1) were fully crossed with three levels of UVBR dose (low: 1 day and approximately 2.5 kJ m^−2^ UVBR, medium: 4 days and approximately 10 kJ m^−2^ UVBR, and high: 8 days and approximately 20 kJ cm^−2^ UVBR; Table S1). To decouple dose and irradiance, the daily time interval of exposure was adjusted for each irradiance treatment so that daily dose remained constant (8, 4 and 1 h of exposure per day for the low, medium and high irradiance treatments, respectively). These UVBR exposures were centred on midday and administered in addition to 12 h light:12 h dark non-UVBR background lighting. It is worth noting that the primary aim of the acute UVBR exposure regimes employed in this study was not to represent field exposures, but rather to investigate the long-term impacts of short-term UVBR exposures, with a defined temporal gap between exposure and the physiological effects being measured, so that the extent and severity of true carryover effects could be elucidated.

Temperature during and after the UVBR exposure period was cycled daily to represent summer conditions at the site of collection, ranging from 21 to 31°C. Water temperature of each treatment was measured during the experiment using waterproof iButton temperature data loggers and ranged from 21.5 to 29°C. Additional temperature fluctuations associated with heat emitted by the UVBR lights were minimised by placing trays of ice on ‘buffer shelves’ immediately underneath the high irradiance UVBR treatment during exposure events.

**Fig. 1.**
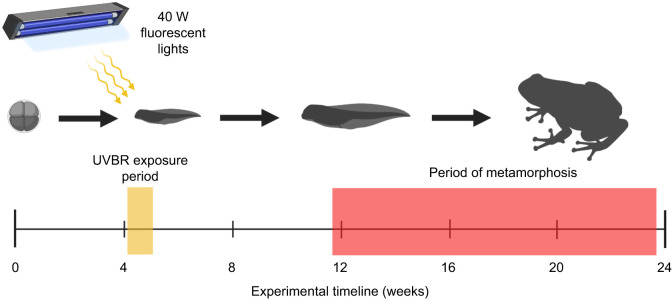
**A schematic diagram of the experimental timeline (in weeks).** The yellow bar on the timeline represents the period of exposure of *Litoria caerulea* larvae (Gosner stage 25) to ultraviolet-B radiation (UVBR) treatments, whilst the time period in which metamorphosis occurred is demonstrated by the red bar. The amphibian silhouettes are for illustrative purposes only. Created with BioRender.com.

After acute UVBR treatment exposures, larvae were reared to metamorphosis as described above (Fig. 1). Fifty animals survived the larval phase and successfully metamorphosed for use in this study (see Lundsgaard et al., 2021, for details on the larval component of the experiment). There was no mortality post-metamorphosis. Age (from egg), size (mass, snout-to-vent length, tibiofibular length and interorbital distance) and body condition (scaled mass index) were measured at metamorphosis (defined as the full reabsorption of the tail at Gosner stage 46). Growth of juvenile frogs in the first month post-metamorphosis was also assessed. One month after metamorphosis, juvenile frogs were tested for jumping performance and foraging efficiency. Upon completion of tests, frogs were euthanised in a MS-222 bath and carcasses were immediately stored at −80°C for analysis of relative telomere lengths (see ‘Traits’, below, for more details).

### UVBR exposures

UVBR conditions were generated using 1.2 m, 40 W fluorescent light tubes (no UVBR: zero bulbs; low irradiance: two bulbs at 51 cm; medium irradiance: six bulbs at 27 cm; high irradiance: eight bulbs at 12 cm; Repti-Glo 10.0, Exo Terra, Montreal, QC, Canada). UVBR and UVAR levels at the water surface of each container were measured using a radiometer/photometer (IL1400BL, International Light Inc., Newburyport, MA, USA) to ensure consistent levels across experimental shelves.

Fluorescent lights are a suitable substitute for natural sunlight when housing reptiles and amphibians, emitting important biologically active wavelengths across the ultraviolet (UV), visible and infrared spectra (Baines et al., 2016). That said, the physiological effects of artificial lighting may differ to sunlight exposure because of differences in spectral composition, warranting caution when extrapolating lab-based results of UVBR-induced physiological effects to the field (Baines et al., 2016). Daily, natural fluctuations of UVBR and UVAR irradiances are correlated (Schuch et al., 2015b), so the ratio of UVBR:UVAR was kept as similar as possible between treatments. UVBR levels used in this study are much lower than ambient UVBR levels measured in the region (500 µW cm^−2^ in air at water level at midday in Brisbane, QLD, Australia; van Uitregt et al., 2007), to help account for attenuation by cloud cover, vegetation cover and dissolved organic matter that reduce aquatic UVBR levels in the wild (Palen et al., 2002; Palen and Schindler, 2010; Olker et al., 2013; Alton and Franklin, 2017).

### Traits

#### Age, size, growth and condition at metamorphosis

Developing larvae were monitored daily, with age at metamorphosis defined as the number of days between egg laying and full reabsorption of the tail (Gosner 46). Juvenile frogs were weighed and photographed on the day of metamorphosis and again 1 month later, allowing for calculations of post-metamorphic growth. Snout-to-vent length, leg length (tibiofibular bone) and interorbital distance (length between the eyes) were determined from photographs (iPod touch 5th generation, Apple) of the dorsal surface of each frog (with a ruler for scale) using the software program ImageJ (National Institutes of Health, Bethesda, MD, USA).

Of the three linear size metrics measured, interorbital distance correlated most strongly with body mass and was therefore used in mass/length calculations of body condition, as suggested by Peig and Green (2009). Condition factor of juvenile frogs was calculated using the scaled mass index (SMI) method developed by Peig and Green (2009), which has been demonstrated as the most suitable and accurate condition index across a range of taxa (Peig and Green, 2010; Brodeur et al., 2020). In accordance with Brodeur et al. (2020), who confirmed the applicability of this method for use in amphibians, we defined the scaling exponent *b* through a non-linear power function regression [mass=α(interorbital distance)*^b^*] for the sample population (minus two outliers) which was: γ=0.0012*x*^2.9624^ (*R*^2^=0.793), to obtain size-independent SMI values. This scaling exponent conforms with allometric scaling observed in other taxa, which typically ranges between 2.5 and 3.2 (Green, 2001). Two outliers did not fit the mass/length trend of the sample population (*n*=50) and were therefore removed from calculation of the regression, following Peig and Green (2009). SMI was calculated relative to the average interorbital distance of the sample population (7.32 mm), giving the estimated mass that each frog (including the two outliers) would have at this fixed body size. Larger SMI values thus indicate larger energy reserves and provide an effective estimate of body condition (Peig and Green, 2009).

#### Foraging efficiency

Four weeks post-metamorphosis, and following a 4 day fasting period, juvenile frogs were tested for indices of foraging efficiency. Individual frogs were placed under a clear lid in the middle of a rectangular foraging arena (17×30×10 cm) for a 5 min adjustment period prior to testing. A cricket of known mass (7–34 mg; average cricket to frog mass ratio=1:27, range=1:104–1:10) was then placed under a holding container at the opposite end of the arena so that the frog was directly facing the cricket. Both holding containers were then lifted simultaneously and the frog was allowed to freely hunt the cricket in the hunting arena. The hunt was timed and recorded using a GoPro Hero 5 (at 120 frames s^−1^ and 1080p) and was terminated the moment the frog captured and swallowed the prey, or in some instances ‘gave up’ on the hunt as a result of fatigue (defined as the point in time in which the frog stopped demonstrating stalking behaviour for 30 s or more). Frogs were then returned to the middle of the arena, placed under a lid, and rested for 5 min. The procedure was repeated two more times (three hunts per day), and these foraging efficiency tests were repeated again 4 days later, for a total of six hunts per frog.

Videos were played back (Tracker Video Analysis and Modelling Tool, Open Source Physics) frame-by-frame by digitising the snout to determine the distance and speed of successful strikes (leading to prey capture). Only the greatest strike distance and strike speed achieved by each frog were compared in statistical analyses. In most cases, but not all, the maximum distance and speed were achieved in the same successful strike. Additionally, the number of strike attempts and duration of prey pursuit until capture were averaged across the six hunts per frog. Two animals (one from the low irradiance, medium dose treatment and one from the low irradiance, high dose treatment) were unresponsive to the presence of prey during all six hunts (instead attempting to escape the foraging arena) and were excluded from analysis. Of the 46 frogs analysed statistically for foraging efficiency, 38 initiated stalking/hunting behaviour in all six trials while the remaining eight animals had either one or two trials excluded from analysis because of a lack of stalking/hunting behaviour.

#### Jumping performance

Four weeks post-metamorphosis, and 2 days after the first foraging efficiency test, the same juvenile frogs were tested for jumping performance. Frogs were placed on top of laminated grid paper and filmed immediately (GoPro Hero 5, 240 frames s^−1^ at 720p) while maximum escape behaviour was being elicited. Frogs that remained stationary were urged to jump by gentle contact to the vent. After at least five jumps, frogs were returned to the middle of the grid paper, placed under a lid, and rested for 5 min. The jump tests were then repeated once more. As with the foraging efficiency trials, recordings were played back (Tracker Video Analysis and Modelling Tool, Open Source Physics) frame-by-frame to determine the distance and speed of jumps. The greatest distance and speed achieved by each frog was considered as the maximum jumping performance of the animal and was analysed statistically. In most cases, but not all, the maximum distance and speed were achieved in the same jump. If frogs jumped farther or faster in a foraging efficiency trial, then these jumps were considered as the maximum jumping performance of the animal and used in the jumping performance analysis.

#### Relative telomere length by qPCR

The qPCR-based relative telomere length assay followed Burraco et al. (2017). Approximately 10 mg (6–17 mg) of hind-leg muscle tissue was dissected (on ice) from each juvenile frog carcass (*n*=2–8 per treatment). Genomic DNA was extracted from the finely chopped muscle tissue using a PureLink Genomic DNA Mini Kit (Invitrogen, Carlsbad, CA, USA), in accordance with the manufacturer's protocol. DNA concentrations were then determined using a Qubit fluorometer (Invitrogen, cat. no. Q32857) and a Qubit dsDNA Broad-Range Assay Kit (Invitrogen, cat. no. Q32853), in accordance with the manufacturer's protocol. DNA samples were aliquoted and stored at −80°C until use.

The broadly conserved nature of telomeric sequence repeats in vertebrates allowed us to use the same primer set as Burraco et al. (2017), i.e. F: 5-AACCAGCCAAGTACGATGACAT-3′ and R: 5′-CCATCAGCAGCAGCCTTCA-3. Species-specific quantitative PCR (qPCR) primers against the glyceraldehyde 3-phosphate dehydrogenase (*GAPDH*) housekeeping gene were designed using PrimerQuest (Integrated DNA Technologies, Coralville, IA, USA) with acceptance of the default parameters, except that amplicon length was set to 95–105 bp. The GAPDH primers were: F: 5′CGGTTTGTTTGGGTTTGGGTTTGGGTTTGGGTTTGGGTT-3′, and R: 5′-GGCTTGCCTTACCCTTACCCTTACCCTTACCCTTACCCT-3′. qPCR was performed using iTaq Universal SYBR Green Supermix (Bio-Rad Laboratories Inc.). We used a 1:40 dilution of DNA samples to suit both the telomere and *GAPDH* primer amplification rates. qPCR for telomere and *GAPDH* genes were performed on separate plates, but in corresponding wells for each sample. We used 1.75 ng of genomic DNA per reaction, and a primer concentration of 500 nmol l^−1^ in a 20 µl reaction containing 10 µl 2× SYBR Green Supermix. PCR cycles for amplification of telomeric repeats were 5 min at 95°C, and then 30 cycles of 1 min at 56°C and 30 s at 95°C. For GAPDH, the 5 min incubation at 95°C was followed by 40 cycles of 60°C for 1 min and 95°C for 30 s. All qPCR cycles were conducted on a Bio-Rad CFX Connect Real-Time System. The efficiency of each qPCR plate performed was determined from a standard curve by serially diluting a combined pool of all the samples (4-fold dilutions to 1:1024 concentration, and corresponding to 70.6, 17.65, 4.413, 1.103 and 0.276 ng of DNA per well). Samples were run in duplicate, and each assay included a separate standard curve as well as a no-template control (in triplicate) at the start and end of the plate (to detect any contamination associated with pipetting).

All PCR efficiencies were above 95%, and unique dissociation curves with single melting peaks were produced, indicating single-product amplification. There was low-range amplification in no-template control wells (Ct value of 30, relative to telomere Ct values of approximately 16.3) despite contingencies in place to prevent contamination. The left-shifted melting peak of no-template control amplification most likely indicates primer-dimerism that should not affect relative telomere length evaluation (Vasilishina et al., 2019). Data were collected using Bio-Rad CXF Manager software (version 3.1), and results were exported to Excel. Relative telomere length was quantified from averaged Ct values in accordance with Vasilishina et al. (2019) by normalising to the reference DNA sample: ΔΔCt=ΔCt_sample_−ΔCt_reference_, where ΔCt=Ct_telomere_–Ct_GADPH_ for each sample. ΔΔCt values were used in statistical analysis and fold-change in telomere length relative to the reference DNA sample was calculated for graphical display, where fold-change=2^−ΔΔCt^ (Pfaffl, 2001).

### Statistical analysis

The no-UVBR treatment proved detrimental to larval fitness for this species (Lundsgaard et al., 2021), resulting in insufficient metamorphs for statistical analysis (*n*=2). Therefore, the no-UVBR treatment was excluded from analyses and multi-factorial comparisons were made between the different UVBR irradiance and dose treatments. Statistical analyses were performed using R version 3.4.2 (*Short Summer*; http://www.R-project.org/), with statistical significance set at 0.05. Egg clutch was included as a random effect, except in models for foraging efficiency metrics and relative telomere lengths, where it did not account for significant variation in data. All models were assessed for suitability of assumptions with diagnostic residual plots. Data are presented as means±s.e.m., unless otherwise stated.

Treatment-specific effects on the progression of metamorphosis over time was assessed with a Cox mixed effects survival analysis utilising the *coxme* (https://CRAN.R-project.org/package=coxme), *Matrix* (https://CRAN.R-project.org/package=Matrix) and *survival* (https://CRAN.R-project.org/package=survival; Therneau and Grambsch, 2000) packages. Treatment-specific differences in average age, body mass and SMI at metamorphosis were modelled with separate linear mixed effects (LME) models (*lmerTest* package, function *lmer*; Kuznetsova et al., 2017). The correlation between age and mass at metamorphosis was also assessed with a simple linear regression.

Following Alton and Franklin (2012), the correlation matrix of body mass, snout-to-vent length, interorbital distance and leg length was assessed with a principal components analysis (PCA) to investigate treatment-associated differences in overall body size at metamorphosis. Of the resulting morphological variables generated, principal component (PC) 1 accounted for 79.43% of the variation, and PC2 accounted for 11.17% of the variation. The PC factor scores for PC1 were then modelled with an LME model. PC2 did not explain sufficient variation to be of particular interest (Quinn and Keough, 2002), and so was not assessed. To assess treatment-associated differences in the growth of juvenile frogs in terms of overall body size, a PCA was performed using the same morphological measurements at 1 month post-metamorphosis. PC1 accounted for 86.28% of the variation and was used in analysis, while PC2 did not explain sufficient variation to be retained for further analysis (7.02%). The PC1 factor scores were modelled with an LME model with the PC1 factor scores from the initial size-at-metamorphosis data included as a covariate.

The data for prey capture time and average and maximum strike attempts until prey capture were skewed to the right and did not satisfy the normal distribution assumption and were therefore modelled with negative binomial generalised linear models (*Mass* package, function *glm.nb*; Venables and Ripley, 2002), with frog mass included as a covariate. Cricket mass was also included as a covariate in the analysis of maximum strike attempts until prey capture. Maximum distance and speed for both jumping performance and successful prey strikes were analysed with LME models. Frog leg length (tibiofibular length) was included as a covariate for jumping performance models, while frog mass was a better predictor in models of maximum strike distance and speed. ΔΔCt values (a proxy for telomere length) were also modelled with an LME model, with larval growth rate (mg per day), frog mass and frog age included as covariates. Tukey contrasts were conducted *post hoc* with adjustment for multiple comparisons using the *multcomp* (function *glht*; Hothorn et al., 2008) and *emmeans* packages (function *emmeans*; https://CRAN.R-project.org/package=emmeans).

## RESULTS

**Fig. 2.**
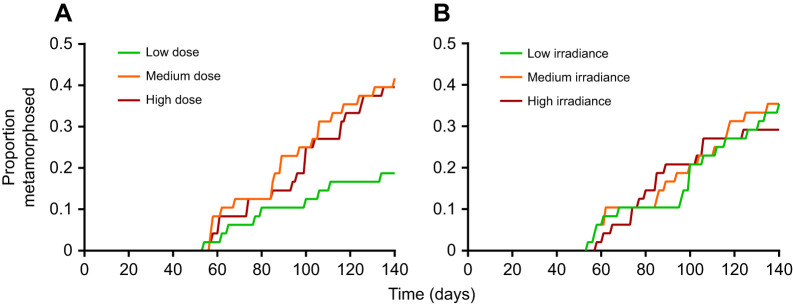
**Effect of UVBR dose and irradiance on the progression of metamorphosis in *L. caerulea*.** The proportion of metamorphosed larvae is shown over a 140 day period following commencement of UVBR (A) dose and (B) irradiance treatments. No interaction between dose and irradiance was present, so data for the dose treatments are pooled across irradiances, and vice versa, to observe main effects (*n*=48 per treatment level).

The survival analysis revealed a significant effect of UVBR dose on the progression of metamorphosis of *L. caerulea* larvae over time (χ^2^_2_=7.742, *P*=0.021), with less than half as many metamorphs developing in the low dose treatments compared with the medium and high dose treatments (*Z*=2.170, *P*=0.076; *Z*=2.602, *P*=0.025, respectively; Fig. 2A). However, there was no significant difference in average age at metamorphosis of these individuals (*F*_2,37_=0, *P*=1). There was no interaction between UVBR dose and irradiance on progression to, or average age at, metamorphosis (χ^2^_4_=3.785, *P*=0.436; *F*_4,36_=2.012, *P*=0.113, respectively), and no significant main effect of UVBR irradiance on these metrics (progression of metamorphosis: χ^2^_2_=4.970, *P*=0.083; Fig. 2B; age at metamorphosis: *F*_2,37_=1.316, *P*=0.281).

**Fig. 3.**
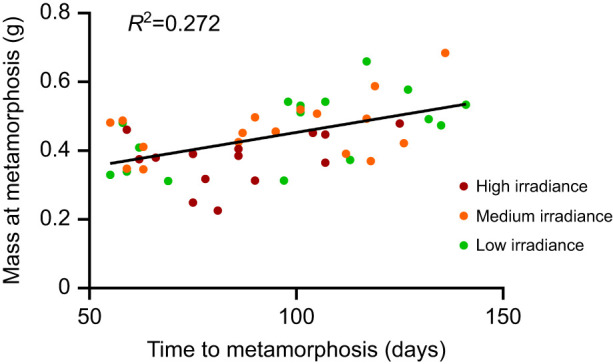
**Correlation between age at metamorphosis and mass at metamorphosis of *L. caerulea* larvae.** Data points represent individual animals, colour-coded by UVBR irradiance treatment (*n*=14–17 per irradiance, pooled across UVBR doses). The trend line indicates a positive correlation between time to metamorphosis and mass (*R*^2^=0.272).

**Fig. 4.**
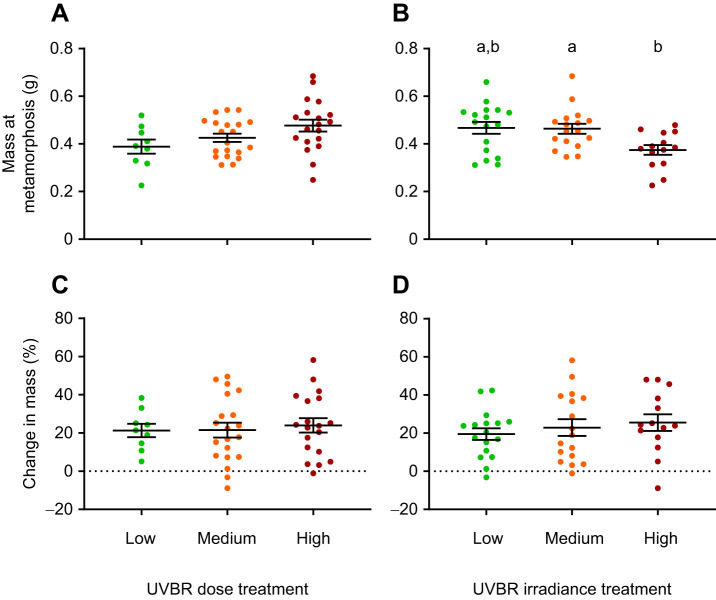
**Effect of UVBR dose and irradiance on mass at metamorphosis and growth after metamorphosis in *L. caerulea*.** Mass (A,B) and growth (percentage change in mass after 30 days, with no net change in mass indicated by the grey dotted line; C,D) are shown following UVBR dose (left) and irradiance (pooled; right) treatments. Data are presented as means±s.e.m. (*n*=9–20 metamorphs per pooled treatment level), and lowercase letters denote significant differences (*P*<0.05) between treatment groups.

There was a positive correlation between age and mass at metamorphosis (*F*_1,46_=17.16, *P*<0.001, *R*^2^=0.272; Fig. 3), with age at metamorphosis a strong predictor of metamorph mass in the LME model (*F*_1,37_=12.384, *P*<0.001). Given that UVBR irradiance and dose did not significantly affect age at metamorphosis (see previous paragraph), we justified the removal of this covariate from analysis to prevent confounding effects with mass. There was a significant effect of UVBR irradiance on mass and overall size at metamorphosis (mass: *F*_2,34_=3.973, *P*=0.028; overall size: *F*_2,39_=3.456, *P*=0.042), whereby larvae exposed to high irradiance UVBR metamorphosed with 20% less mass, on average, than larvae exposed to the low and medium irradiance treatments (*t*=2.290, *P*=0.070; *t*=2.500, *P*=0.044, respectively; Fig. 4B). There was no interactive effect of UVBR dose and irradiance on mass or overall size at metamorphosis (*F*_4,33_=0.920, *P*=0.464; *F*_4,39_=0.735, *P*=0.574, respectively), and no main effect of UVBR dose on these metrics (mass: *F*_2,34_=1.231, *P*=0.304; overall size: *F*_2,39_=2.091, *P*=0.137; Fig. 4A). Size at metamorphosis predicted body size in the month following (*F*_1,38_=96.867, *P*<0.001), but there was no significant effect of larval UVBR treatment on growth during this time (dose: *F*_2,37_=0.305, *P*=0.739; Fig. 4C; irradiance: *F*_2,37_=0.784, *P*=0.464, Fig. 4D; interaction: *F*_4,36_=1.203, *P*=0.326).

**Fig. 5.**
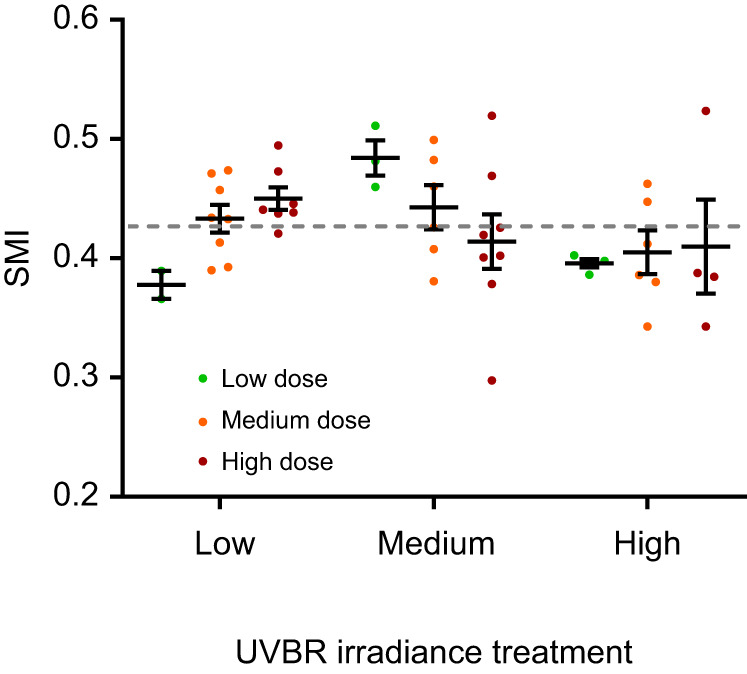
**Effect of UVBR dose and irradiance on body condition of *L.*** ***caerulea*****.** Scaled mass index (SMI) values represent the estimated mass when scaled to the mean interorbital distance of the sample population. Data are means±s.e.m. (data points represent individual animals, *n*=2–8 per treatment). The grey dashed line represents the mean SMI of the sample population, such that points above the line indicate good relative body condition, while points below the line indicate poor relative body condition.

There was a significant interaction between UVBR dose and irradiance on juvenile frog body condition (interaction: *F*_4,36_=2.797, *P*=0.040), whereby the effect of UVBR dose depended on the irradiance at which it was administered. High UVBR dose exposure improved metamorph body condition when administered at a low irradiance but reduced body condition when administered at medium and high irradiance (Fig. 5). Larvae exposed to high irradiance UVBR had reduced body condition at metamorphosis regardless of the dose administered, with an average 7.6% reduction in mass when scaled for the average interorbital distance of the sample population, compared with the low and medium irradiance treatments. Because of these interactive effects, the low dose UVBR treatment yielded the greatest differences in body condition depending on the irradiance of administration, with larvae that received a low UVBR dose metamorphosing with the worst SMI when administered at a low irradiance, but the best SMI when administered at a medium irradiance (*t*_35_=−2.856, *P*=0.019; Fig. 5).

**Fig. 6.**
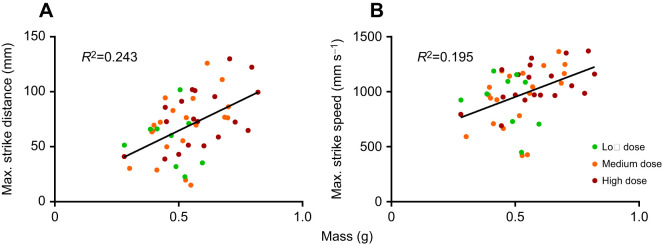
**Correlation between mass and foraging performance of *L. caerulea*, 1 month post-metamorphosis.** (A) Maximum distance and (B) speed of successful prey capture strikes. Data points represent individual animals, colour-coded by UVBR dose treatment (*n*=9–20 per dose, pooled across UVBR irradiances). Trend lines indicate a moderate positive correlation between mass and foraging performance.

There was a significant effect of frog mass on foraging efficiency as measured by maximum successful strike distance and strike speed (*F*_1,37_=13.338, *P*<0.001; *F*_1,37_=9.435, *P*=0.004, respectively), with larger frogs able to jump farther and faster when striking prey (Fig. 6). However, there was no significant effect of larval UVBR exposure on foraging efficiency of subsequent juvenile frogs, as measured by foraging time (dose: χ^2^_2_=0.408, *P*=0.816; irradiance: χ^2^_2_=0.383, *P*=0.826; interaction: χ^2^_4_=2.241, *P*=0.692), average number of strike attempts (dose: χ^2^_2_=1.573, *P*=0.455; irradiance: χ^2^_2_=0.449, *P*=0.799; interaction: χ^2^_4_=0.450, *P*=0.978), maximum number of strike attempts (dose: χ^2^_2_=1.718, *P*=0.424; irradiance: χ^2^_2_=0.215, *P*=0.898; interaction: χ^2^_4_=1.098, *P*=0.895), maximum successful strike distance (dose: *F*_2,37_=0.995, *P*=0.379; irradiance: *F*_2,37_=1.151, *P*=0.327; interaction: *F*_4,37_=1.795, *P*=0.151) and maximum successful strike speed (dose: *F*_2,37_=0.409, *P*=0.667; irradiance: *F*_2,37_=1.274, *P*=0.292; interaction: *F*_4,37_=1.066, *P*=0.387; Table 1). Cricket size did not influence maximum strike attempts until successful prey capture (χ^2^_1_=0.081, *P*=0.775).

**Table 1.**
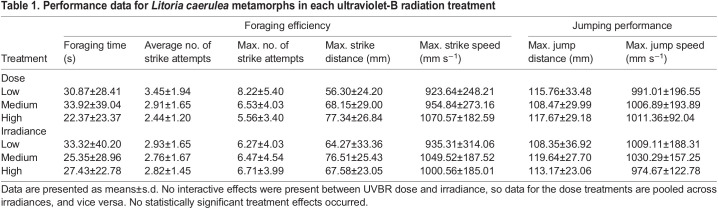
Performance data for *Litoria caerulea* metamorphs in each ultraviolet-B radiation treatment

There was a significant effect of frog size (especially tibiofibular length) on jumping performance as measured by maximum jumping distance and maximum jump speed (jumping distance: *F*_1,37_=7.789, *P*=0.008; jumping speed: *F*_1,38_=13.442, *P*<0.001), with larger frogs able to jump farther and faster (data not shown). However, there was no significant effect of larval UVBR treatment on metamorph jumping distance (dose: *F*_2,33_=0.130, *P*=0.879; irradiance: *F*_2,32_=2.099, *P*=0.139; interaction: *F*_4,33_=1.113, *P*=0.367) and maximum jumping speed (dose: *F_2,38_*=0.377, *P*=0.689; irradiance: *F*_2,38_=0.250, *P*=0.780; interaction: *F*_4,38_=0.202, *P*=0.936; Table 1).

**Fig. 7.**
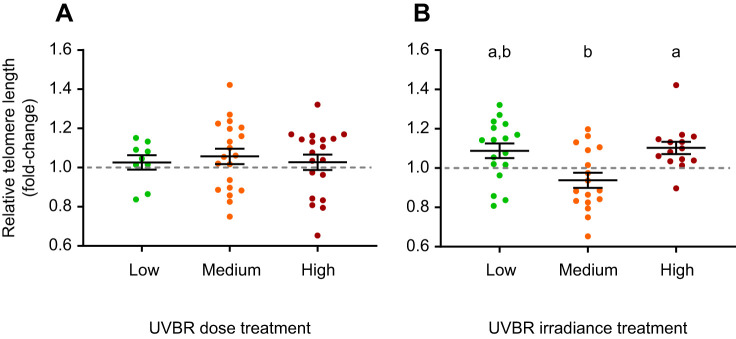
**Effect of UVBR dose and irradiance on relative telomere length of *L.*** ***caerulea*** **1** **month post-metamorphosis.** UVBR (A) dose and (B) irradiance treatment effects, are shown as a fold-change from the reference sample (a pool of all samples, indicated by the grey dashed line). Data are presented as means±s.e.m. (*n*=9–20 per pooled treatment level), and lowercase letters denote significant differences (*P*<0.05) between treatment groups.

UVBR irradiance had a significant effect on relative telomere length (*F*_2,39_=6.427, *P*=0.004; Fig. 7B), with juvenile frogs exposed to medium irradiance UVBR as larvae having shorter relative telomere lengths as frogs than those previously exposed to high irradiance UVBR (*t*_39_=2.711, *P*=0.026), but not low irradiance UVBR (*t*_39_=−2.061, *P*=0.112). There was no interaction between larval UVBR dose and irradiance on relative telomere length (*F*_4,39_=0.477, *P*=0.752), and no main effect of UVBR dose on this metric (*F*_2,39_=0.610, *P*=0.548; Fig. 7A). Frog age and mass did not have a significant effect on relative telomere length (*F*_1,35_=0.124, *P*=0.726; *F*_1,38_=0.874, *P*=0.356, respectively), nor did larval growth rate (*F*_1,35_=0.031, *P*=0.861). However, the two juvenile frogs in the no-UVBR treatment had some of the longest relative telomere lengths (1.27 and 1.55 times the average relative telomere length of the sample population).

## DISCUSSION

In this study, acute UVBR exposure of *L. caerulea* larvae (Gosner stage 25) produced detrimental carryover effects that transcended metamorphosis, impacting size and body condition in the juvenile stage, which is associated with reduced fitness and survival in the field (Chelgren et al., 2006; Scott et al., 2007). Only a few other studies have explicitly demonstrated carryover effects of UVBR exposure in amphibians through metamorphosis (Pahkala et al., 2001; 2003; Ceccato et al., 2016). Pahkala et al. (2001) found that embryos exposed to an enhanced dose of UVBR suffered from a higher prevalence of developmental abnormalities as larvae, were delayed in metamorphosis, and were smaller as juveniles compared with animals that were not exposed to UVBR. Ceccato et al. (2016) found that a 6 week exposure to UVBR in the larval phase led to immune system changes in subsequent juvenile frogs. In all these cases, the detrimental effects of UVBR were not evident in the life-history stage being exposed, but instead manifested later in life (Pahkala et al., 2001; Ceccato et al., 2016). These contrasting effects of early-life UVBR exposure on different life stages is evidence for a life-history trade-off that may be driven by changes in energy balance, a phenomenon that has been documented in other taxa (Debecker et al., 2015). It is possible that the increased energy requirements for somatic maintenance in animals exposed to high irradiance UVBR as larvae may have led to reduced energy reserves being available at metamorphosis (Alton et al., 2012), as evidenced by lower SMI values in this study. Our work corroborates the pervasive nature of UVBR-induced carryover effects in amphibians through metamorphosis, highlighting the importance of tracking fitness consequences of UVBR exposure into later life-history stages, as the predicted impacts of UVBR on amphibian populations may otherwise be underestimated.

Contrary to hypotheses, the medium and high UVBR dose treatments significantly improved progression into metamorphosis compared with the low dose treatment, an effect mainly driven by reduced mortality throughout the larval phase. High UVBR doses can also improve growth, development and swimming performance in the larval stage of this species (Lundsgaard et al., 2021). These results may reflect increased endogenous vitamin D_3_ in larvae exposed to high UVBR doses, a vitamin essential for maintaining calcium homeostasis, which in turn affects bone mineralisation, muscular function and nerve function (Stiffler, 1993; Antwis and Browne, 2009; Michaels et al., 2015). It is possible that a deficiency in calcium stores hindered successful metamorphosis in animals that did not receive sufficient UVBR exposure, because of the need for increased bone formation during this time (Stiffler, 1993).

Post-metamorphosis, a deficiency in vitamin D_3_ can lead to nutritional metabolic bone disease, characterized by a weakened skeleton (Densmore and Green, 2007). Importantly, juvenile frogs in this study were fed crickets and cockroaches that were themselves reared on carrots and dry cat food containing essential vitamins and minerals, so it is unlikely that the juvenile frogs were in a nutrient-deficient state. This could explain why there was no mortality post-metamorphosis, and why there were no effects of UVBR treatment on jumping performance and foraging efficiency of juvenile frogs in the present study. Still, larval exposure to high irradiance UVBR caused reduced size at metamorphosis, which in turn was correlated with reductions in some foraging efficiency and jumping performance metrics. This result provides further evidence of the detrimental fitness consequences of reduced size at metamorphosis (Chelgren et al., 2006; Scott et al., 2007).

The mechanisms driving the somewhat opposing effects of UVBR dose and irradiance on juvenile *L. caerulea* health are not known. However, it seems that certain post-metamorphic benefits conferred by a high dose of larval UVBR exposure were only expressed at lower, more tolerable irradiances. This was particularly apparent with the SMI data. Although caution is warranted in interpretation given the small sample size, our data show a positive trend between metamorph body condition and UVBR dose when administered at a low irradiance. At medium irradiance, the beneficial effects of UVBR exposure were only conferred at a low and medium dose, whilst any amount of exposure to high irradiance UVBR proved detrimental to metamorph body condition. These results suggest that the effect of a given UVBR dose are highly dependent on the irradiance at which it is administered, which highlights the importance of the rate of DNA damage production (determined by irradiance) for physiological outcomes of UVBR exposure (Pandelova et al., 2006; Londero et al., 2019). It seems that even large doses of UVBR exposure can be managed by *L. caerulea* if irradiance, and thus the rate of formation of pyrimidine dimers in DNA, is low. If however, UVBR irradiance is great enough that the rate of DNA damage exceeds the rate of DNA repair (dose–toxicity threshold), then an accumulation of DNA damage is expected (Pandelova et al., 2006). This accumulation of damage, or the rate at which it is induced, along with the energy required to repair it, could explain the subsequent detrimental effects of the high irradiance UVBR treatments in later life-history stages (Alton et al., 2012; Debecker et al., 2015). In fact, UVBR-induced DNA damage can even induce permanent molecular changes including epigenetic modifications and telomere shortening in exposed animals, all of which are expected to generate physiological carryover effects (O'Connor et al., 2014; Young, 2018).

The relative telomere lengths of the two frogs that were not exposed to UVBR were much greater than the average telomere length of the UVBR-exposed sample population in this study, suggesting that this stressor can shorten telomeres in developing *L. caerulea* post-metamorphosis. However, further research with suitable sample sizes is required to confirm this finding. It is known that UVBR-induced DNA lesions in the telomeric sequence can cause telomere shortening directly (Stout and Blasco, 2013; Ikeda et al., 2014). However, this is the first study to our knowledge that has demonstrated a telomere shortening carryover effect in tissues that were not directly exposed to UVBR, but rather developed post-exposure in juvenile frogs (hind-leg muscle). It is not known whether a potential ‘latent’ effect of DNA lesions on telomere shortening exists, but this question warrants further investigation.

Contrary to hypotheses, it was the medium irradiance treatment that induced the shortest relative telomere lengths. Our results support those of Burraco et al. (2017), who also found that larvae that metamorphosed larger and with more fat reserves had shorter telomeres, reflecting increased metabolic rate, metabolic-induced ROS production, and cell division in these animals (Savini et al., 2013; Burraco et al., 2017; Young, 2018). Given that frogs in the high irradiance UVBR treatment had poorer short-term fitness but longer relative telomere lengths than larvae exposed to medium irradiance UVBR, our findings support the notion that a trade-off exists between immediate/short-term fitness gains and long-term health consequences in amphibians responding to environmental stress (Burraco et al., 2017). The apparent non-monotonic relationship between UVBR irradiance and relative telomere length (Fig. 7B) has likely resulted because UVBR can affect multiple counteracting drivers of telomere length (Vandenberg et al., 2012). For example, high irradiance UVBR is expected to induce the greatest oxidative stress (Heck et al., 2003; Kazerouni et al., 2016), but also the least growth and cell division, which have opposing effects on telomere length (Young, 2018). This could explain why animals in the low and high UVBR irradiance treatments had similar relative telomere lengths despite different UVBR exposure regimes. Further research elucidating the complex interactions between stressors that cause telomere shortening but also impact variables that counteract telomere shortening (e.g. reduced growth) are warranted.

*Litoria caerulea* larvae experienced relatively high mortality rates across all treatments, a caveat of this study that presents opportunities for future research. A deprivation of vitamin D_3_ is the simplest and most likely explanation for treatment-specific differences in larval survival and future studies should consider administering a chronic UVBR exposure regime throughout the larval period that better reflects natural rearing conditions for this species, as opposed to a no-UVBR reference treatment (Michaels et al., 2015). The described rearing conditions in two studies by Cabrera-Guzman et al. (2011, 2013) suggest that *L. caerulea* larvae were able to metamorphose successfully under relatively low UVBR provisioning. Importantly, these larvae were reared on a diet of algal pellets and other organic matter and may have obtained their necessary vitamin D_3_ and calcium requirements through food intake (Antwis and Browne, 2009; Cabrera-Guzman et al., 2011; 2013). The feeding regime in the present study has been used to successfully rear other amphibian species (including *Limnodynastes terraereginae* and *Limnodynastes peronii*), but it is plausible that a diet of spinach, which has no vitamin D_3_ and contains oxalates and vitamin A that can reduce calcium absorption (Johansson and Melhus, 2001; Sorensen, 2014), contributed to the lethal and sub-lethal effects observed. The possibility that the egg clutches collected were in poor condition also cannot be ruled out. Further research into the UVBR requirements of larval amphibians in the presence and absence of dietary vitamin D_3_ and calcium supplementation would improve understanding of the conditions under which UVBR exposure can benefit larval development.

The finding that an acute exposure to UVBR in the larval period can impact indices of short-term and long-term fitness post-metamorphosis has implications for our understanding of how this stressor may be shaping amphibian population dynamics. Studies of UVBR exposures in South and Central America suggest a doubling to tripling in the frequency of short-term maximum UVBR exposure events in relatively recent times as a result of changes in the distribution of ozone and cloud cover (Middleton et al., 2001; Schuch et al., 2015a). It is likely that similar increases in acute UVBR events will continue to occur in some tropical regions due to climate change (Hegglin and Shepherd, 2009; Mckenzie et al., 2011; Williamson et al., 2014). Although perturbations to early life-history stages of amphibians may not drive population declines as much as previously assumed (Vonesh and De la Cruz, 2002; Vonesh, 2005), the present study highlights an additional mechanism by which UVBR exposure in early-life-history stages could shape population dynamics, by way of carryover effects into the juvenile stage. An integrated approach incorporating mesocosm and field studies that bridge the gap between laboratory and field data is needed for determining the potential role that UVBR-induced physiological carryover effects play in amphibian population declines. Importantly, dose and irradiance must be jointly considered when taking UVBR field measurements to elucidate the impacts of this stressor on amphibian populations, to account for the complex interactive effects between these UVBR parameters on amphibian health.

## Supplementary Material

Supplementary information
