# Supplementary material for: Early exposure to UV radiation causes telomere shortening and poorer condition later in life
Source: J Exp Biol. 2022 Sep 2;225(17):jeb243924. doi: 10.1242/jeb.243924 (PMC9482364; doi:10.1242/jeb.243924)
Supplement: Supplementary information [file jexbio-225-243924-s1.pdf]

Table S1. Ultraviolet-B and ultraviolet-A irradiances (μW cm^{-2}) and cumulative dose estimates (kJ m^{-2}) for each treatment

**Exposure duration (days)**	**Ultraviolet-B**		**Ultraviolet-A**	
	**Irradiance (μW cm^{-2})**	**Dose (kJ m^{-2})**	**Irradiance (μW cm^{-2})**	**Dose (kJ m^{-2})**
**1**	Low (8.7 ±0.6; 8h/day)	2.508 ±0.160	Low (39.6 ±4.2; 8h/day)	11.397 ±1.211
	Medium (35.3 ±2.4; 2h/day)	2.540 ±0.169	Medium (205.1 ±17.3; 2h/day)	14.766 ±1.245
	High (70 ±1.6; 1h/day)	2.522 ±0.059	High (470.0 ±36.1; 1h/day)	16.920 ±1.299
**4**	Low (8.7 ±0.6; 8h/day)	10.032 ±0.640	Low (39.6 ±4.2; 8h/day)	45.588 ±4.844
	Medium (35.3 ±2.4; 2h/day)	10.160 ±0.676	Medium (205.1 ±17.3; 2h/day)	59.064 ±4.98
	High (70 ±1.6; 1h/day)	10.088 ±0.236	High (470.0 ±36.1; 1h/day)	67.680 ±5.196
**8**	Low (8.7 ±0.6; 8h/day)	20.064 ±1.280	Low (39.6 ±4.2; 8h/day)	91.176 ±9.688
	Medium (35.3 ±2.4; 2h/day)	20.320 ±1.352	Medium (205.1 ±17.3; 2h/day)	118.128 ±9.960
	High (70 ±1.6; 1h/day)	20.176 ±0.472	High (470.0 ±36.1; 1h/day)	135.36 ±10.39

Animals in the no-UVBR control group were reared in the absence of UVBR. Measurements were taken using a radiometer/photometer (IL1400BL, International Light Inc., Newburyport, USA) at the water surface of each of the containers. Dose estimates were calculated based on the UVBR irradiance measurements. Values are presented as mean ± standard deviation. These data are also presented in Lundsgaard et al. (2021) which reported on an earlier phase of this larger experiment.

Journal of Experimental Biology • Supplementary information
